# Can Innovation Agglomeration Reduce Carbon Emissions? Evidence from China

**DOI:** 10.3390/ijerph18020382

**Published:** 2021-01-06

**Authors:** Jianqing Zhang, Haichao Yu, Keke Zhang, Liang Zhao, Fei Fan

**Affiliations:** 1 Institute of Central China Development, Wuhan University, Wuhan 430072, China; jqzhang@whu.edu.cn (J.Z.); hcyyyy@whu.edu.cn (H.Y.); zhangkk@whu.edu.cn (K.Z.); 2 Institute of Regional and Urban-Rural Development, Wuhan University, Wuhan 430072, China; 3 School of Economics and Management, Wuhan University, Wuhan 430072, China; 4 School of Business, Hubei University, Wuhan 430072, China

**Keywords:** innovation agglomeration, energy intensity, carbon emission, dynamic spatial Durbin model

## Abstract

Innovation agglomeration plays a decisive role in improving the input–output scale and marginal output efficiency of factors. This paper takes carbon emissions as the unexpected output and energy consumption as the input factor into the traditional output density model. The dynamic spatial panel Durbin model is used to analyze the mechanism for innovation agglomeration and energy intensity to affect carbon emissions from 2004 to 2017 in thirty Chinese provinces. Then, we test the possible mediating effect of energy intensity between innovation agglomeration and carbon emissions. The major findings are as follows. (1) The carbon emission intensity has time-dependence and positive spatial spillover effect. That is, there is a close correlation between current and early carbon emissions, and there is also a high-degree correlation between regional and surrounding areas’ carbon emissions. (2) Carbon emissions keep a classical inverted U-shaped relation with innovation agglomeration, as well as with energy intensity. However, the impact of innovation agglomeration on carbon emissions in inland regions of China does not appear on the right side of the inverted U-shaped curve, while carbon emissions are subject to a positive nonlinear promoting effect from energy intensity. (3) When the logarithm of innovation agglomeration is more than 3.0309, it first shows the inhibition effect on energy intensity. With the logarithm of innovation agglomeration exceeding 5.0100, it will show the dual effect of emission reduction and energy conservation. (4) Energy intensity could work as the intermediary variable of innovation agglomeration’s influence on carbon emissions. Through its various positive externalities, innovation agglomeration can produce a direct impact on carbon emissions, and through energy intensity, it can also affect carbon emissions indirectly.

## 1. Introduction

Material wealth has been greatly increased amid the rapid expansion of urbanization and global industrialization, but meanwhile, the consumption of a large amount of energy in the extensive model of economic development has brought severe challenges to the ecological environment, such as the greenhouse effect and the rising of sea level increased with global climate change. Such serious ecological and environmental problems have attracted more and more attention from governments [1]. While carbon emissions account for the most of the greenhouse gas caused by anthropogenic activities [2], global carbon emissions are expected to increase by 30% above the 2010 level by 2030 [3]. At present, the problem of carbon emissions is becoming increasingly serious, threatening the well-being of mankind.

Given the impact of climate change and global warming, carbon emission reduction has become a hot topic worldwide and has attracted extensive attention from many countries. C. Watanabe (1995) appealed to the industry to replace limited fossil energy with clean energy, based on the 20-year development experience of renewable energy in Japan [4]. G.A. Efthimeros (2000) believed that improving energy efficiency was the key to improving the industrial competitiveness of EU countries, which could not only significantly reduce the operating costs of industrial enterprises, but also reduce the carbon emission caused by economic activities [5]. In addition, China [6], Thailand [7], Britain [8], and other countries’ relevant scholars discussed the energy production and consumption of each country, and all advocated that industrial sectors replace fossil energy with renewable energy to reduce carbon emissions in the manufacturing process.

Technological innovation, as a significant factor contributing largely to the enhancement of energy efficiency, is an important way to realize low-carbon economy, emission reduction, and energy conservation [9,10]. Existing research on technological innovation and carbon emission is mainly carried out from three aspects. (1) Based on EKC curve, those papers study the relationship between technological innovation, economic growth, and carbon emissions. With the improvement of income level and the enhancement of environmental awareness, technological innovation not only plays a role in economic development, but also takes environmental protection into account [11,12]. Especially, low-carbon technological innovation is of great significance to carbon emission reduction [13]. (2) The driving factors of carbon emissions was explored by the IPAT equation and STIRPAT model [14]. The results show that technological innovation is helping to slow down the growth of carbon emissions. (3) The impact of technological innovation on carbon emissions in different industrial sectors has been frequently reported to play a huge role in energy conservation and emission reduction in agriculture [15], transport [16], electrical engineering [17], construction industry [18], and automobile industry [19].

However, technological innovation was found by Acemoglu et al. (2012) to have two-sided effects on carbon emissions [20]. As we all know, technological innovation can reduce carbon emission through using clean energy, adopting carbon capture and storage technology, and improving energy efficiency [21,22]. On the other hand, while promoting economic scale growth, technological innovation might cause more carbon emissions by require higher energy consumption. For example, Jin et al. (2014) [23], according to the empirical data analysis of 35 industrial sectors in China, found that the effect of emission reduction brought by technological efficiency improvement cannot offset the effect of carbon emission growth caused by labor efficiency improvement. Additionally, this labor efficiency improvement was due to economic growth promoted by technological efficiency improvement. Furthermore, Fisher-Vanden et al. (2004) [24] believed that advanced technology could reduce energy consumption intensity. However, due to the “rebound effect”, the output level was greatly increased; thus, the total energy consumption and carbon emission increased. Yan et al. (2011) [25] used the data sample of China’s manufacturing industry and found that in the short term, the technological innovation of manufacturing was conducive to reducing environmental pollution, but in the long term, there was no inevitable causal relationship between technological innovation and environmental pollution. Therefore, carbon emission and technological progress have an uncertain relationship.

China’s economy has been growing dramatically since the reform and opening up. Nevertheless, China is forced to reduce carbon emissions, due to the great pressure brought by the extensive development of traditional industries with “low efficiency, high consumption, and high input”. As indicated by the International Energy Agency (IEA), as a result of the rapid increase of total emission, China is ranked first throughout the whole world in terms of carbon emissions [26]. Under the premise that promoting low-carbon economy and tackling climate change have become a consensus worldwide, the Chinese government announced in 2020 to achieve carbon neutrality by 2060 and proposed that carbon emissions should peak by 2030. The proposal of achieving energy conservation and emission reduction brings challenges to China’s future economic development, and also provides important opportunities for China’s green economic transformation. As a critical driving force in economic and social development, technological innovation will provide strong support for China’s goal of reducing emissions and saving energy.

As for China’s reality, with the rise of the urban agglomeration economy, innovation activities and achievements have a strong regional cluster characteristic. Abundant studies proved that technological progress is an important method to achieve energy conservation and emission reduction in China [27]. Technological progress can contribute to an improvement in carbon emission efficiency, found by Li et al. (2020) [28]. Xue et al. (2020) [29] also found that the direct effect of technological innovation was significantly negative in carbon emission. However, Shen et al. (2014) [30] investigated the relationship between spatial agglomeration and carbon emission reduction from a new economic geographical perspective. They found that the externality of spatial agglomeration is an important mechanism to reduce carbon emission, and spatial agglomerations of different levels and ways correspond to different carbon emission behaviors. On the one hand, as a compact spatial economic behavior, innovation agglomeration is accompanied by various positive externalities and has significant spatial spillover effect. It enables the subject of innovation to acquire tacit and explicit knowledge, and greatly reduces the cost of innovation, so as to enhance the level of innovation output and improve the utilization efficiency of elements, showing the positive role of reducing emissions and saving energy. On the other hand, innovation agglomeration may accelerate energy consumption and carbon emission by expanding regional production scale and factor input. This will have a negative impact on the target of reducing emissions and saving energy. However, the traditional agglomeration theory holds that there are a variety of spillover effects from agglomeration, and the spatial concentration of elements is conducive to saving various costs and improving the efficiency of production [31]. The agglomeration process, when environment and energy are considered to be input factors of production, should contribute to the enhancement of the efficiency of environmental elements and energy [32]. Therefore, can innovation agglomeration show the double effect of reducing emissions and saving energy as predicted by theory? According to China’s reality analysis, will innovation agglomeration lead to increased regional energy consumption and aggravated environmental damage? China has a vast territory, and provinces have significantly different resource endowments, development stages, and other conditions. What are the differences between different regions in terms of the effect of innovation agglomeration on carbon emissions?

As for the method, existing studies showed that pollution was not a purely local environmental problem [33]. Wesley Burnett et al. (2013) [34] estimated the relationship between U.S. state-level carbon emissions by a long panel data, economic activity, and other factors, exploring several spatial models to account for spatial dependence between states. Therefore, spatial autocorrelation should be taken into account to research carbon emission intensity [35,36], and there are already some applications in China [37,38]. Therefore, a spatial econometric model can be used to analyze the spatial effect, considering the “path-dependence” of carbon emission intensity, ignoring the influence of time effect and space effect will lead to systematic bias.

From the above analysis, the existing literature has carried out a relatively rich discussion on the relationship between energy, technological innovation, and carbon emissions. However, there are still relatively insufficient theoretical and empirical studies on integrating the three into the same analysis framework. Additionally, most of the current studies in China only focus on the inhibition of innovation on carbon emission, but ignore the possible promoting effects. Meanwhile, the differences of the effects of innovation on reducing energy consumption and carbon emissions have not been deeply studied. Based on the foregoing, this paper introduces energy factors and carbon emissions into output density model, using 2004–2017 panel data samples from thirty Chinese provinces. The spatial dynamic econometric model is used to test the relationship among innovation agglomeration, energy intensity, and carbon emission, which can control both spatial and endogenous effect. The marginal contribution of this research is threefold: (1) The output density model proposed by Ushifusa and Tomohara (2013) was extended in this paper [39]. We put energy and carbon emission into the output density model, and mathematically explain the effect and mechanism of innovation agglomeration and energy intensity on carbon emission under different threshold conditions. (2) We discuss the possible double effects of energy conservation and emission reduction that innovation agglomeration may exhibit, and tests the possible mediating effect of energy intensity between innovation agglomeration and energy intensity. (3) The dynamic panel Durbin model combined with Han Phillips (GMM) [40] estimation method is used to control the time lag effect, spatial lag effect, and endogenous problem of carbon emission, which provides reliable empirical support for the proposed theoretical hypothesis under robust conditions.

The remaining parts of this paper are arranged in the following manner: The second part constructs the theoretical model, normatively exploring the effect of innovation agglomeration on energy conservation and emission reduction. An empirical model is established in the third part to explain the data sample. The fourth part discusses and analyzes the empirical results. Finally, conclusions and recommendations are given.

## 2. Theoretical Model and Research Hypothesis

Innovation agglomeration refers to the intensity of human innovation activities in unit space. It is generally believed that regions with a higher degree of innovation agglomeration have a larger output scale in the same space. The aggregation of innovative activities in different spaces can bring about various spillover effects through sharing, matching, and learning [41,42]. Different from the traditional neoclassical model, the output density model proposed by Ciccone and Hall (1996) allows for increasing returns to scale, and this model fully considers the influence of spatial factors on output, thus providing a basic analytical framework for depicting spatial production activities [43]. Ushifusa and Tomohara (2013) [39] further simplified the output density model as follows:

(1) $$q_{i}=Q_{i}/A_{i}=\tau_{i}{\left[{\left(n_{i}\right)}^{\beta }k_{i}^{1-\beta }\right]}^{\alpha }{\left[Q_{i}/A_{i}\right]}^{(\lambda -1)/\lambda }$$

where *Q_i_* and *A_i_* are respectively non-farm output and total area of the region; *q_i_* is the unit area non-agricultural output of the *i* region, namely the output density; *τ_i_* is the Hicks neutral parameter; *n_i_* is employment density; β(0 < β < 1) represents the income share of labor to capital input; and *k_i_* is the capital input per unit area. The main difference between Equation (1) and traditional Cobb–Douglas production function is that it contains the parameter α(0<α≤1) representing the income share of the two inputs, capital and labor, to land. It reflects the diminishing marginal productivity of the factors caused by crowding. The smaller α represents the lower factor production efficiency [39]. Ciccone and Hall (1996) [43] identified the loss of productivity due to additional factor input per unit area as congestion effect, and give the following explanation: Under the condition of Hicks neutral technology, land elements are relatively fixed. With the increased input of capital and labor, factor input will deviate from the optimal configuration levels of capital–land and labor–land. This gradually reduces the marginal output level and factor productivity of capital and labor per unit area, leading to diminishing marginal returns of capital and labor. Obviously, α=1 means the marginal return of the factor is constant. *λ*(*λ* > 1) refers to the output coefficient of density. (*λ* − 1)/λ is output elasticity, which is used to reflect the agglomeration effect. It is clear that the bigger *λ*, the greater the elasticity of output density (*λ* − 1)/*λ*, the stronger the positive externality of agglomeration, and the larger its contribution to output density. Therefore, *λ* can be regarded as the externality parameter of agglomeration. Because of the close link between the agglomeration effect and the stage of innovation development, the spatial concentration of innovation activities in different stages has different agglomeration effects. Usually, regions with a high level of innovation development will also present strong agglomeration externalities [44]. Therefore, the size of *λ* corresponds to different stages of innovation development. More importantly, the existence of *λ* gives the output density function the possibility of showing increasing returns to scale. After sorting out Equation (1), the final form of output density can be obtained as follows:

(2) $$q_{i}=Q_{i}/A_{i}=\tau_{i}{\;}^{\lambda }\left[{\left(n_{i}\right)}^{\beta }k_{i}^{1-\beta }\right]{}^{\alpha \lambda }$$

It can be seen that the size of *αλ* determines the scale return characteristic of output density function. There is 0 < *α* ≤ 1, but as long as λ is large enough to make *αλ* > 1, then the output density can show the feature of increasing returns to scale. At this point, the contribution of positive externalities of agglomeration effect to output is greater than the “inhibition” effect of crowding effect on output, which makes up for the loss of production efficiency caused by element agglomeration in a given space. It is obvious that the output density function reflects the crowding effect of production factors and the agglomeration effect of innovation activities simultaneously. It also allows the production function to have different scale return characteristics under different conditions (the relationship between *αλ* and 1). It will provide a general explanation of the real innovation activities from the perspective of spatial behavior.

However, the existing output density model has some limitations. It does not reflect the energy and environmental constraints caused by innovative activities. Given this, this paper tries to incorporate energy and environment (carbon emission) into this model. From the input side, energy, as a basic factor input in the production process, can be included into the production function together with capital and labor [45]. From the perspective of output, pollutants such as carbon emissions can be regarded as a kind of undesired output resulting from the use of energy factors, and could be included into the production function together with the expected output [46]. Therefore, based on the facts in the actual production process and the assumptions of existing studies, this paper takes energy consumption and carbon emissions as input factor and undesired output respectively, and integrates them into the above output density function. Let *E_i_* be the energy consumption and *e_i_=E_i_/A_i_* be the energy consumption per unit area. At the same time, it is assumed that the entire production process will produce Ci units of carbon emissions. In this way, (1) can be extended to:

(3) $$(Q_{i}+C_{i})/A_{i}=(Q_{i}/A_{i})(1+C_{i}/Q_{i})=\tau_{i}{\left[{\left(n_{i}\right)}^{\beta }\kappa_{i}^{l}e_{i}^{1-\beta -l}\right]}^{\alpha }{\left(Q_{i}/A_{i}\right)}^{(\lambda -1)\lambda }(1+C_{i}/Q_{i}{)}^{(\lambda -1)\lambda }$$

where *l* (0 < *β*+*l*< 1) is the contribution rate of innovation capital input to output per unit area, α is redefined as the share of output per unit area of physical capital, labor and energy. The meanings of other variables are consistent with the above. Assuming that capital can free flow across regions. Therefore, the capital price (the interest rate) is equal in every region under the equilibrium. Under the equilibrium, the marginal output of capital in the factor market is equal to the capital price, so the capital demand density (the ratio of total capital *K* to regional area) can be expressed as:

(4) $$\kappa_{i}=\frac{K_{i}}{A_{i}}=\frac{\alpha l}{r}\times (Q_{i}/A_{i})(1+C_{i}/Q_{i})$$

Substituting (4) into (3) to get:

(5) $$1+\frac{C_{i}}{Q_{i}}=\tau^{\frac{\lambda }{1-\alpha l\lambda }}\times {\left(\frac{\alpha l}{r}\right)}^{\frac{\alpha l\lambda }{1-\alpha l\lambda }}\times {\left(\frac{Q_{i}}{N_{i}}\right)}^{\frac{-\alpha \beta \lambda }{1-\alpha l\lambda }}\times {\left(\frac{E_{i}}{Q_{i}}\right)}^{\frac{\alpha (1-\beta -l)\lambda }{1-\alpha l\lambda }}\times {\left(\frac{Q_{i}}{A_{i}}\right)}^{\frac{\alpha \lambda -1}{1-\alpha l\lambda }}$$

The logarithm of both sides of (5) can be obtained as follows:

(6) $$\ln(1+\frac{C_{i}}{Q_{i}})=\phi -\frac{\alpha \beta \lambda }{1-\alpha l\lambda }\ln(\frac{Q_{i}}{N_{i}})+\frac{\alpha (1-\beta -l)\lambda }{1-\alpha l\lambda }\ln(\frac{E_{i}}{Q_{i}})+\frac{\alpha \lambda -1}{1-\alpha l\lambda }\ln(\frac{Q_{i}}{A_{i}})$$

where $\phi =\frac{\lambda }{1-\alpha l\lambda }\ln\tau +\frac{\alpha l\lambda }{1-\alpha l\lambda }(\ln\alpha l-\ln r)$

As a result, (6) can be transformed into:$\ln(1+C_{i}/Q_{i})\approx C_{i}/Q_{i}$

(7) $$\frac{C_{i}}{Q_{i}}=\phi -\frac{\alpha \beta \lambda }{1-\alpha l\lambda }\ln(\frac{Q_{i}}{N_{i}})+\frac{\alpha (1-\beta -l)\lambda }{1-\alpha l\lambda }\ln(\frac{E_{i}}{Q_{i}})+\frac{\alpha \lambda -1}{1-\alpha l\lambda }\ln(\frac{Q_{i}}{A_{i}})$$

The left side of Equation (7) is the carbon emission intensity of non-farm output, and the right side contains the innovation agglomeration level *Q_i_/A_i_*, energy intensity *E_i_/Q_i_,* and labor productivity *Q_i_/N_i_*. It indicates that under the condition of considering innovation agglomeration, carbon emission intensity depends on the density of output, energy intensity, and labor productivity. Among the three, the direction of carbon emission intensity being affected by output density and energy intensity is determined by agglomeration externalities and the return on investment. Therefore, under different levels of agglomeration externalities, the impact of innovation agglomeration and energy intensity on carbon emission intensity is also different. The comparative static analysis results obtained in this paper based on Equation (7) are discussed (Table 1).

### 2.1. Effect of Innovation Agglomeration on Carbon Emission Intensity

When 1 < *λ* < 1/*αl*, the improvement of innovation agglomeration brings no benefit to reducing carbon emission intensity. The economy is in the preliminary phase of innovation at this time. During this stage, the degree of innovation concentration is constantly improving, and the results of innovation output are significantly increased. However, the economies of scale, technology spillover, and knowledge sharing effect brought by innovation agglomeration are still not obvious. Technological innovation is mainly aimed to improve the economic output per unit of labor time [47]. The rapid expansion of production capacity will increase the total amount of pollution emission per unit space and aggravate the environmental pollution, thus leading to a higher level of carbon emission intensity. In addition, to pursue economic benefits, the government has relatively lax environmental regulations and low environmental protection standards, which cannot form an effective “driving force” effect on polluting enterprises [48]. As a result, carbon emission per unit of output continues to increase, so carbon emission intensity is affected by innovation agglomeration in a positive direction.

When *λ* > 1/*α*l, the improvement of innovation agglomeration helps reduce carbon emission intensity. At this time, the economy is in the mature stage of innovation. The agglomeration of a large number of enterprises reduces the risk of technological innovation of a certain individual enterprise. This is beneficial to the exchange, learning, and large-scale promotion of energy conservation and emission reduction technologies among enterprises [49]. In the meanwhile, spillovers and sharing of various green technologies, centralized supervision of environmental departments, and other positive externalities brought by higher innovation agglomeration level can give full play to economies of scale, reduce the cost of environmental supervision and environmental publicity, so as to curb carbon emissions [50]. Meanwhile, innovation can help reset the technological trajectory and trigger a series of subsequent and related innovations. It will lead to the forming of new technology and production systems, which could bring a significant decline in carbon emissions [51]. For example, the introduction of battery technology in the automobile industry has led to a series of innovations that have reduced the reliance of automobiles on fossil energy. In addition, due to the continuous improvement of technological level, the tertiary industry has an increasing share in the market due to its low-level carbon emissions and low-level energy consumption. The industrial structure has been adjusted to the direction of the tertiary industry. The green upgrading effect of regional industrial structure brought by innovation agglomeration has somewhat prevented carbon emission intensity from increasing [52]. Based on this, the direct impact of innovation agglomeration on carbon emission intensity mainly depends on the direction of technological progress and the comprehensive effect of positive or negative externalities of agglomeration.

Therefore, this paper proposes the theoretical Hypothesis 1.

**Hypothesis** **1** **(H1):** *With the improvement of innovation agglomeration level, carbon emission intensity will show an inverted U-shaped trend when other conditions remain unchanged.*

### 2.2. Influence of Energy Intensity on Carbon Emission Intensity

The accumulation of regional innovation resources is still in the initial and accelerated stage when 1 < *α* < 1/*αl*. Meanwhile, the innovation output level is gradually improving but still relatively low. The consumption of fossil energy is usually regarded as the direct source of environmental pollution in environmental economics. The combustion of fossil fuel represented by coal will directly produce carbon dioxide, sulfur dioxide, and other pollutants in production process [53]. In this period, the externality of agglomeration has not been fully reflected, and the increase of energy intensity has no positive effect on reducing carbon emission. Although the regional innovation level has been improved, the innovation output of most enterprises is mainly based on the introduction, imitation, and re-absorption of technology in developed regions, and their independent innovation ability is relatively weak [54]. From the point of empirical fact, some developing countries, due to the low prices of labor, energy, land rent, and other factors, and the lax environmental regulations, attracted a lot of high-pollution and high-energy-consumption industries from developed countries [55]. Although they could absorb some of the international technology spillovers, they also become “pollution haven” of foreign investment. At the same time, it is difficult to make breakthroughs in technologies for reducing emissions and saving energy in a short period, bringing a certain degree of time lag. Additionally, the high cost of new energy development makes it difficult to achieve the price advantage. Therefore, the energy consumption structure dominated by traditional fossil energy is difficult to change [15]. This problem makes it hard to effectively decouple carbon emission intensity and energy intensity, and may even hinder the reduction of carbon emission intensity.

When *λ* > 1/*αl*, the region is in the mature stage of innovation development. The higher level of innovation agglomeration enables its positive externalities to be significantly manifested, and the improvement of energy intensity is beneficial for reducing carbon emission intensity. The various spillover effects of innovation agglomeration and characteristics of economies of scale become prominent in this stage. It will effectively promote the concentrated use and improve the use efficiency of energy products in production and consumption [56]. The breakthrough of new energy technology can effectively reduce the production cost and market price of clean energy, and therefore the need for clean energy and new energy will increase significantly. This is also useful for promoting the “green” adjustment of energy consumption structure [57]. Above all, the direct impact of energy intensity on carbon emission intensity mainly depends on the structure of energy consumption. More fossil fuel combustion will inevitably lead to an increase in carbon emission intensity. The use of clean and new energy from innovation agglomeration not only substitutes part of fossil fuel, but also optimizes the energy consumption structure, which has a certain inhibition effect on carbon emission. On this basis, theoretical Hypothesis 2 is proposed:

**Hypothesis** **2** **(H2):** *With the increase of energy intensity, carbon emission intensity will show an inverted U-shaped trend when other conditions remain unchanged.*

### 2.3. The Mediating Effect of Energy Intensity between Innovation Agglomeration and Carbon Emission Intensity

Through the above analysis, it is found that innovation agglomeration, energy intensity, and carbon emission are closely related. Innovation agglomeration is the main manifestation of spatial allocation of various innovation elements and spatial distribution of innovation activities. The latter will undoubtedly have an important impact on energy demand and carbon emissions [58] and also have an important impact on production efficiency of energy elements [59]. From this perspective, the direct impact of innovation agglomeration on carbon emissions is mainly reflected in various spillover effects of agglomeration, such as agglomeration of innovative enterprises, which benefits the learning and advancement of green innovation technologies [60]. Carbon emission usually comes from fossil energy combustion. Therefore, once innovation agglomeration has an impact on energy utilization efficiency, it is bound to have an indirect impact on carbon emission. On the one hand, the invention and use of new and clean energy are conducive to reduce energy consumption. It will decline the per unit of energy consumption output and improving utilization efficiency [61]. On the other hand, agglomeration can improve the efficiency of production factors by technology spillover, knowledge sharing, and factor matching, so as to achieve the improvement of energy utilization efficiency [62]. Thus, innovation agglomeration exerts an indirect influence on carbon intensity by influencing energy efficiency. Accordingly, this paper further puts forward theoretical hypothesis 3.

**Hypothesis** **3** **(H3):** *Energy intensity may have a mediating effect in the impact of innovation agglomeration on carbon emission intensity.*

## 3. Data Description and Empirical Model

### 3.1. Data Description

#### 3.1.1. Explained Variable: Carbon Emission Intensity (CO_2_)

The carbon emission calculation method of IPCC (2006) [63] is adopted in this paper. The energy types considered are all the 17 fossil energy sources appearing continuously in the China Energy Statistical Yearbook, including natural gas, refinery dry gas, washing coal such as washed coal, briquette, and coal, coking products like coke oven gas and coke, and oil products like kerosene liquefied petroleum, fuel oil, diesel oil, crude oil, and gasoline [64].

**Figure 1:**
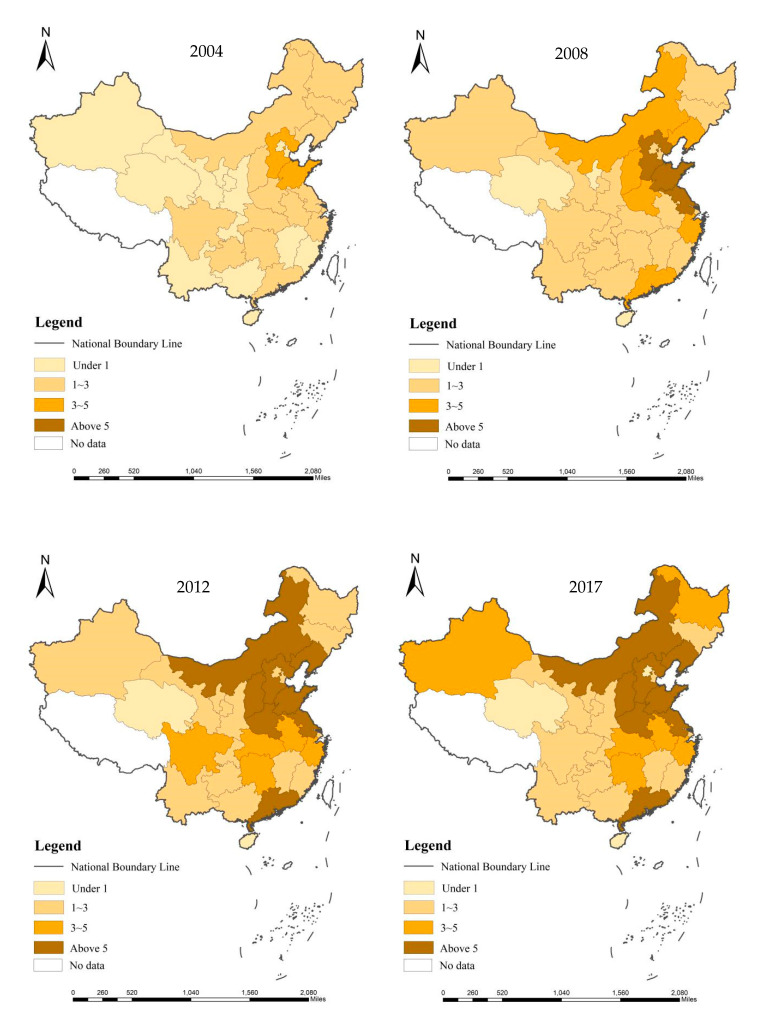
China provincial carbon emission intensity from 2004 to 2017.

From the comparison of different years, it can be found in Figure 1 that from 2004 to 2017, the carbon emission intensity in China at the provincial level has been continuously enhanced, showing the characteristics of contiguous distribution. The eastern coastal region is ranked first and the central region second in terms of carbon emission intensity. In the western region, only Inner Mongolia has relatively high carbon emission intensity. It also can be seen that in the aspect of carbon emission intensity, various regions have a relatively strong spatial correlation, and there is a phenomenon of pollutant diffusion between regions. Therefore, the spatial econometric model can be used in this paper to explore the influence of innovation agglomeration at the provincial level on carbon emission intensity.

#### 3.1.2. Core Explanatory Variables

(1) Innovation Agglomeration (*Agin*): Existing literature mainly uses Taylor coefficient of innovation activities [65], location entropy [66], and spatial Gini coefficient [67] and other indicators to measure innovation concentration. This paper is consistent with the theoretical model. Referring to the measurement method of output density model [39], this measurement of innovation agglomeration can describe the spatial density and distribution of innovation activities. Patent data are often used because of their palpable link with innovation output and their relative integrity [68]. In this paper, the number of patents granted (pieces) is selected to represent regional innovation output. The specific calculation formula is as follows:

(8) $$A{\mathrm{gin}}_{it}=In_{it}/S_{i}$$

where the subscripts *i* and *t* correspond to different provinces and years, respectively; *In_it_* refers to the number of patents granted of province *i* in year *t*, *S_i_* represents the administrative area of province *i.*

(2) Energy Intensity (*Sen*): Consistent with the theoretical model, the ratio of total energy consumption to industrial value-added (10^4^ yuan) is used in this paper to measure energy intensity.

The formula is as follows:

(9) $$S{\mathrm{en}}_{it}=E_{it}/Y_{it}$$

where *Y_it_* is the industrial value-added of province *i* in year *t*, and the energy intensity (*Sen_it_*) is the energy consumption level of unit non-farm output.

#### 3.1.3. Control Variables

(1) Labor productivity (*Lab*): Consistent with the theoretical model, measured by the ratio of non-farm output to the number of employees. An increase in labor productivity could lead to an increase in carbon emissions [69]. (2) Per capita income level (*Pcin*): Measured by the natural logarithm of GDP per capita. According to the classical EKC hypothesis [70], in this paper, the first power term *Pcin* and the second power term *sPcin* of per capita GDP derived from natural logarithms are introduced into the model. (3) Urbanization (*Ur*) is the ratio of urban population to total population. On one hand, a large amount of energy will be consumed with the acceleration of urbanization, increasing carbon emission [71]. On the other hand, with the improvement of urbanization level, carbon emission is reduced under the low-carbon green city development mode in which new environmental protection technologies are applied extensively [72]. In this paper, the first power term *Ur* and the second power term *sUr* are introduced into the model at the same time. (4) The ratio of coal consumption to total energy consumption is used to measure energy consumption structure (*Es*). Coal still occupies a dominating position in China’s energy consumption structure, and carbon emission intensity is lower when the proportion of coal consumption is higher. (5) Industrial structure (*Ind*): Expressed as the ratio of industrial value-added to GDP. It is harder to reduce carbon emission intensity when industry accounts for a larger proportion in the economic structure [73]. (6) Opening up (*Open*): Measured by the proportion of total import and export in GDP. A higher degree of openness can help a region to better absorb green technology spillover and advanced management experience from advanced countries, contributing to energy conservation and emission reduction [74]. (7) Environmental regulation (*Er*): Measured by the proportion of environmental expenditure in GDP [75]. Higher environmental regulation intensity will force enterprises to carry out technological innovation and choose clean energy to reduce carbon emission intensity. (8) Marketing (*Mar*): Fan Gang’s marketization index [76] was used to measure the marketing degree. Innovative elements would enjoy free flow when there is a higher level of marketing, but the impact of marketing on carbon emission is uncertain.

### 3.2. Data Source

The panel data, obtained on the basis of availability from 30 provincial administrative regions (provinces, municipalities and autonomous regions) (Tibet was excluded due to serious data deficiency) in China from 2004 to 2017, are used as research samples in this paper. The main sources and explanations of sample data are shown in Table 2. Among them, all kinds of monetary quantity indexes are deflated by the prices in 2000. For non-percent variables, this paper uses their natural logarithms to reduce the degree of dispersion of sample data.

### 3.3. Empirical Model

#### Test Model for the Effect of Innovation Agglomeration and Energy Intensity on Carbon Emission Intensity

Based on above analysis, carbon emission intensity is featured with a strong spatial correlation in China. Without considering the inherent spatial spillover effect, measurement results may be biased [77]. The spatial correlation of different sources can be well reflected by the spatial Durbin model. Therefore, this paper mainly adopts the spatial Durbin model to carry out empirical test.

(10) $$\begin{array}{l} CO_{2}{}_{\mathrm{it}}=\beta_{0}+\beta_{1}CO_{2}{}_{i,t-1}+\rho_{1}\sum_{i=1}^{n}w_{ij}CO_{2}{\;}_{jt}+\beta_{2}Ag{\mathrm{in}}_{i,t}+\beta_{3}sAg{\mathrm{in}}_{i,t}+\rho_{2}\sum_{i=1}^{n}w_{ij}Agin{\;}_{jt}+\\ \beta_{4}{\mathrm{Sen}}_{i,t}+\beta_{5}s{\mathrm{Sen}}_{i,t}+\rho_{3}\sum_{i=1}^{n}w_{ij}Sen{\;}_{jt}+\delta \sum X_{it}+\lambda \sum_{i=1}^{n}w_{ij}X{\;}_{jt}+\;\varepsilon_{it}+u_{it} \end{array}$$

Carbon emission changes may have time-dependence, namely time lag effect, and carbon emission may have a two-way causal relationship with technological progress, energy efficiency, and other factors, which may cause endogeneity [78]. Considering the above reasons, in this paper, the one-phase lag of carbon emission is introduced into the standard static space panel Durbin model. Based on (7), build dynamic spatial panel Durbin model as follows:

Where CO_2_ is the carbon emission intensity, *i* stands for province, and *t* stands for year; *CO_2__(t−1__)_* represents the carbon emission with one-phase lag, which is used to control and investigate the time lag effect of carbon emission intensity; *Sen_it_* denotes energy intensity and *Agin_it_* refers to the degree of innovation agglomeration. In addition, this paper introduces the quadratic term of innovation aggregation (*sAgin_it_*) and the quadratic term of energy intensity (*sSen_it_*) into the model, trying to test Hypothesis 1 and Hypothesis 2. Considering the limitations of using only economic matrix or geographical matrix, this paper uses the geographical and economic distance spatial weight matrix. *ρ* and *γ* refer to the spatial lag coefficient of each main explanatory variable and control variable, respectively. *β1~β5* are the coefficients to be evaluated, in which *β1* is the time lag coefficient of carbon emission intensity, *ε* is random disturbance term, and *u* represents regional fixed effect.

### 3.4. Parameter Estimation Method

Because Equation (10) includes spatial lag terms and time lag terms of explained variables as explanatory variables, the residual no longer satisfies the basic assumptions of homoscedasticity and exogeneity. Traditional ordinary least square (OLS) method, fixed effect and random effect estimation would get biased estimation results, and the most commonly used method in estimating the stunned spatial panel Durbin model, maximum likelihood estimation (MLE), is also powerless to control the potential endogeneity. The spatial generalized method of moments (GMM) proposed by Han and Phillips (2010) [40] can effectively overcome the problem of weak instrument in traditional instrumental variable method and difference GMM estimation. Additionally, its restrictions on the sample section number N and time T are weak. Particularly, under the condition of small sample, this method can still get a consistent and unbiased estimation result. Therefore, this method is mainly adopted in the following part. For comparison, we also report the estimation results under non-spatial panel OLE-FE, non-spatial panel SYS-GMM, and static spatial Durbin panel model.

## 4. Empirical Results and Discussion

### 4.1. Spatial Correlation Test Results

From the results reported in Table 3, it can be obviously seen that the Global Moran’s I corresponding to each spatial regression equation is significant at 1% level, and LM-lag and LM-error statistics are both significant. This indicates that the explained variables of each equation have obvious spatial correlation. Therefore, it is necessary to research the problems in this paper by the spatial Durbin panel model, which cannot degenerate to spatial lag model or spatial error model. Considering the regional individual differences and the estimation bias caused by temporal factors, the dynamic spatial panel model with spatiotemporal bidirectional fixed effect is mainly used in this paper to estimate the parameters. It is more effective to choose fixed effect model when only some specific individuals are involved in the regression analysis. Moreover, Hausman test results also show that the panel regression model in this paper is suitable for estimation considering fixed effect (due to the limited space, this paper does not report the Hausman test results. Interested readers can obtain the results from the author).

### 4.2. Impact of Innovation Agglomeration and Energy Intensity on Carbon Emission—An Empirical Test of Hypothesis 1 and Hypothesis 2

#### 4.2.1. Regression Results

From Table 4, the coefficients of most control variables in Model 1 without considering endogeneity and spatial correlation are not significant, and the coefficients of innovation agglomeration and its quadratic term in Model 2 where spatial correlation is not considered are not significant. They indicate that bias may appear if spatial correlation is ignored. The estimation results of Model 3 and Model 4, which take spatial correlation into consideration, indeed show better statistical characteristics. Further, from the contrast of Model 3 and Model 4, the significance level of most control variables and core explanatory variables in Model 4 is better than that in Model 3. Additionally, in Model 4, the coefficient of time lag term of carbon emission is positive at a significant level of 1%, which verifies the carbon emission intensity change described above has a time-dependence feature. The time lag effect, spatial correlation, and endogeneity of the explained variables are taken into account in Model 4. Therefore, compared with the other three models, it has the best performance in measurement and theoretical expectation. Based on this, this paper would focus on the estimation results of dynamic spatial panel Durbin model (Model 4) with GMM estimation method in the following discussion.

According to Table 4, carbon emission intensity has significant space spillover effect and time lag effect simultaneously. The coefficient of time lag term of carbon emission intensity is significantly positive at 1%, showing that there is a strong path-dependence in carbon emission intensity in time dimension. Obviously, it shows a “snowball effect”. If there is a high level of carbon emission intensity in the last year, in the next year it may also go up continuously. On the other hand, the Model 4′s spatial coefficient is significantly positive at 1%, indicating that carbon emission intensity among regions has a palpable spatial agglomeration phenomenon. Therefore, a certain spatial spillover effect can be detected. A region’s carbon emission intensity is closely related to that of neighboring regions.

Both the first and second power coefficients of innovation agglomeration are significant at 1% level, and the sign of the former is positive while the latter is negative. It indicates the existence of an obvious negative correlation between innovation agglomeration and carbon emission intensity. For a quadratic function, referring to Woodridge (2002) [79], the threshold value can be defined as the turning point of the function or the variable value when the function gets the maximum value (minimum value). First, when the natural logarithm of innovation agglomeration level is below 5.0100, the value of inflection point, it has significant promoting effect on carbon emissions. In this period with economic developing rapidly, innovation elements quickly gather in the region, and rich innovative activities cause large economic output and carbon emissions. Meanwhile, the application of technological achievements also has a certain time lag. The positive externalities of energy conservation and emission reduction technologies have not yet appeared. Thus, there is obvious increase in carbon emission intensity, if the natural logarithm of innovation agglomeration level exceeds 5.0100, and begins to show inhibiting effects on carbon emission intensity. Due to the spillover effects of innovation agglomeration, such as positive externalities, cost saving and specialized division of labor, emission reduction and energy conservation begin to appear. The gathering of a large number of enterprises is conducive to sharing facilities for saving energy and reducing emissions, minimizing the risk of applying “green innovation” technology. From the present stage of each province, in 2017, the innovation agglomeration level of most China’s provinces exceeded the inflection point, which shows that improving the level of innovation agglomeration helps cut down carbon emission intensity in most provinces and then it is beneficial for China’s overall carbon emission reduction. Only those of Inner Mongolia, Yunnan, Qinghai, Gansu, and Xinjiang are below the inflection point. It indicates that the agglomerations of innovation achievements in these provinces mainly take effect in expanding production capacity and improving economic operation efficiency. The technological progresses in these areas are not green-oriented, and they will still face great pressure of cutting down carbon emission in the future.

The quadratic term coefficient is significantly negative at 5% level. In terms of energy intensity, the coefficient is significantly positive at 1% level. It shows that there is a typical inverted U-shaped link between carbon emission intensity and energy intensity. The increase of energy intensity has a synergistic influence on carbon emission intensity if the natural logarithm of energy intensity is lower than the value of inflection point 0.8536. When it exceeds the inflection point, carbon emission intensity is subject to an inhibitory effect from the increase of energy intensity. For this reason, the energy intensity mainly depends on the progress of green innovation technology [31]. When regional innovation agglomeration is at a low level, it is difficult for the positive externalities of agglomeration to promote the improvement of energy efficiency. Meanwhile, the promotion and application of new energy also has a certain lag and the economics of scale of energy consumption has not formed. The increase of energy intensity brought by capacity expansion has also pushed up the carbon emission intensity. When the regional innovation concentration level is relatively high, the knowledge spillover effect brought by innovation will further improve enterprises’ use of technologies for saving energy and reducing emission. Then the energy structure will be adjusted towards a cleaner one, which exposes carbon emission intensity to a restraining effect from the increase of greener energy intensity. The results manifest that Hypothesis 2 proposed above is valid.

As for control variables, a significantly positive relation is found in the estimated coefficient of labor productivity. Carbon emission intensity will increase by 0.4202% as a result of every 1% increase in labor productivity. This indicates that carbon emission reduction is negatively affected by the improvement of labor productivity. The coefficient of per capita GDP is significantly positive, but the corresponding quadratic term coefficient is significantly negative. This proves the existence of a significant inverted U-shaped link between carbon emission intensity and per capita GDP, which verifying EKC hypothesis. The significantly positive first power and second power coefficients of urbanization indicate that carbon emission intensity may be enhanced due to rapid urbanization in China, and the positive externalities of urban innovation agglomeration are not prominent. The coefficient of energy structure is significantly positive. Carbon emission intensity will increase by 0.2191% as a result of every 1% increase in energy structure. It proves again that the current high-carbon energy consumption structure in China has hindered the reduction of carbon emission intensity to a large extent. The significantly positive coefficient of industrial structure also indicates that the reduction of carbon emission intensity is impeded in the industrial structure with a large industrial proportion. The coefficient of opening up is significantly negative, indicating that the introduction of FDI plays a positive role in cutting down carbon emission intensity due to its higher environmental standards and the spillover effect of advanced technologies for saving energy and reducing emission. The significantly negative coefficient of environmental regulation reveals the improvement in the intensity of environmental regulation in China and its effective function in reducing carbon emission. From 2005 to 2009, China experience a reduction of about 48.1% in its carbon intensity, and by the end of 2019, 15.3% of China’s total energy consumption was contributed by non-fossil energy. China has completed ahead of schedule the target of saving energy and reducing emission promised to the international community at the Copenhagen Climate Summit. From the significantly positive coefficient of marketing degree, it can be seen that China’s market economy has been developing rapidly at the cost of destroying the environment. Therefore, in the process of saving energy and reducing emission, the most important thing is to maintain the normal market competition order through government supervision. It will help to make up for the negative externalities caused by market failures, such as waste of resources and environmental pollution, to create a healthy and orderly market environment for carbon emission intensity reduction.

#### 4.2.2. Further Testing by Subregion

China has a vast territory, and there are great differences in economic development level and energy characteristics among different regions. The overall economic development has shown a basic trend from south to north, from inshore to inland [80]. The leading economic development in coastal areas also directly leads to the rapid increase in energy consumption and the continuous emission of carbon dioxide. However, China’s petrochemical energy resources such as coal, crude oil, and natural gas are mainly distributed in inland areas, producing more high energy consumption and high pollution products such as electric power for coastal areas, resulting in an increasing in China’s overall carbon emission intensity. Therefore, based on China’s inshore and inland areas, this paper further discusses the regional differences in the impact of innovation agglomeration and energy intensity on carbon emission intensity.

From Table 5, the spatial correlation coefficient shows that carbon emission has a stronger positive spatial spillover effect in inshore areas, which is stronger than in inland areas, and all of them are significant at the 1% level. It shows that China’s inshore areas have gradually become the gathering places of global factories and domestic exporting enterprises relying on their comparative advantages of labor, land and other factors. However, this extensive growth mode also results in a huge ecological and environmental cost. The path-dependence and “snowball effect” of carbon intensity are still significant in both inshore and inland areas of China.

From the perspective of innovation agglomeration, the first power and second power coefficients of innovation agglomeration in China’s inshore areas are significant, and the signs are positive and negative, respectively. Similar to the regression results of the whole China, innovation agglomeration and carbon emission intensity have a significantly negative correlation. The first power and second power coefficients of innovation agglomeration in China’s inland areas are significantly positive and non-significantly negative, respectively. This indicates that the negative correlation between innovation agglomeration and carbon emission intensity in China’s inland areas is not obvious, which is only limited to the left part of the inverted U-shaped curve. In terms of energy intensity, the first power and second power coefficients in China’s inshore areas are positive and negative, respectively, both significant at 1% level, indicating the existence of a typical inverted U-shaped curve relation between carbon emission intensity and energy intensity. However, it should be noticed that the first power and second power coefficients of energy intensity in China’s inland areas are significantly positive at 1%, showing no negative relation.

For control variables, the directions of their influence on carbon emission intensity in inshore areas are, respectively, the same as those in inland areas, but the significance is different. In inshore areas, the improvement in labor productivity, industrial structure, and degree of marketization has a higher degree of promotion on carbon emissions than inland areas. Urbanization, energy consumption structure in inshore areas significantly increased the carbon emission intensity, while opening up and environment regulation greatly decreased it. Additionally, carbon emissions intensity also shows a significant inverted U-shaped curve relationship with per capita GDP.

The reasons why there is different performance between the inshore and the inland are as follows. First of all, inshore areas have an excellent environment of technology researching and developing leading the country. With the improvement of innovation agglomeration level, the investment of science and technology resources and the turnover of technology market have been increasing rapidly year by year. Meanwhile, the degree of opening up is much higher than the national average. Market economy has a higher degree of freedom. This is more conducive to the imitation, absorption, and re-innovation of internationally advanced technologies for saving energy and reducing emission, promoting the continuous green upgrading of industrial structure. Inshore areas will have a good performance in reducing carbon emission intensity and improving the ecological environment. They are expected to reduce overall carbon emission intensity in China by exerting various positive externalities of the agglomeration effect. For China’s inland areas, the relation between carbon emission intensity and innovation agglomeration stays at the left part of the inverted U-shaped curve. This is because under the unbalanced regional economic development pattern, China’s inland region receives a large number of pollution-intensive enterprises transferred from inshore region, which objectively increases the carbon emission intensity of inland areas. There is a large number of labor, capital, and production activities concentrated in inshore areas, making their carbon emission intensity higher than those of inland areas. Therefore, the elasticity and significance of the estimated coefficients are higher in coastal areas than in the inland.

### 4.3. Energy Intermediation Effect Test Based on Carbon Emission

Based on the theoretical Hypothesis 3, innovation agglomeration may affect carbon emission through energy intensity. In order to test whether energy intensity acts as an intermediary variable, this paper uses a normative mediation effect model to do further empirical research. The specific mediation effect test model is set as follows:

(11) $$\begin{array}{l} S{\mathrm{en}}_{\mathrm{it}}=\eta_{0}+\eta_{1}Sen_{i,t-1}+\theta_{1}\sum_{i=1}^{n}w_{ij}Sen{\;}_{jt}+\eta_{2}Ag{\mathrm{in}}_{i,t}+\eta_{3}sAg{\mathrm{in}}_{i,t}+\theta_{2}\sum_{i=1}^{n}w_{ij}Agin{\;}_{jt}+\\ \varsigma \sum X_{it}+\theta_{3}\sum_{i=1}^{n}w_{ij}X{\;}_{jt}+\;\zeta_{it}+\iota_{it} \end{array}$$

(12) $$\begin{array}{l} CO_{2}{}_{\mathrm{it}}=\alpha_{0}+\alpha_{1}CO_{2}{}_{i,t-1}+\pi_{1}\sum_{i=1}^{n}w_{ij}CO_{2}{\;}_{jt}+\alpha_{2}Ag{\mathrm{in}}_{i,t}+\alpha_{3}sAg{\mathrm{in}}_{i,t}+\pi_{2}\sum_{i=1}^{n}w_{ij}Agin{\;}_{jt}+\\ \varphi \sum X_{it}+\pi_{3}\sum_{i=1}^{n}w_{ij}X{\;}_{jt}+\;\nu_{it}+\sigma_{it} \end{array}$$

Equations (10)–(12) constitute a complete process of the mediating effect test [81]. If the coefficients α, β, and η of innovation agglomeration are significant, and the coefficient β is smaller or significantly smaller than corresponding α, it indicates that there is a mediating effect. At the same time, we will also study the energy conservation effect of innovation agglomeration. Specifically, we need to examine the sign of innovation agglomeration and its quadratic coefficient in Equation (12). If at least one coefficient is significantly negative, it indicates that innovation agglomeration can show energy conservation effect under certain conditions. Similarly, for comparison and robustness test, this paper also gives the estimated fixed effects of the dynamic spatial panel Durbin model, the static spatial panel Durbin model, the non-spatial dynamic panel model, and the non-spatial panel model (Table 5). The main interpretation model is still the dynamic spatial panel Durbin model.

The estimation results in Table 6 show the significantly positive coefficients of the time and spatial lag terms of energy intensity and carbon emission. It indicates that these two variables have significant path-dependence as well as time and space spillover effects. The significantly positive coefficient of spatial lag term of innovation agglomeration also indicates that the innovation agglomeration of neighboring provinces also has a significant impact on energy intensity and carbon emission. In model 15, the coefficient of innovation agglomeration is significantly positive at 10%, and the coefficient of quadratic term is significantly negative at 1%. This shows the existence of a distant negative relation between innovation agglomeration and energy intensity. As long as the logarithm of innovation agglomeration is more than 3.0309, it will show energy conservation effect. In 2017, only in Inner Mongolia, Qinghai, and Xinjiang, the level of innovation agglomeration was lower than 3.0309, who did not enter the stage of inhibiting energy intensity by innovation agglomeration. Obviously, the critical value of energy conservation effect (3.0309) of innovation agglomeration is significantly smaller than that of emission reduction effect of innovation agglomeration (5.0100). With the improvement of innovation agglomeration, it will show the inhibition effect on energy intensity firstly. When it continues to increase to a certain threshold, it will show the dual effect of saving energy and reducing emission. Besides, compared with its emission reduction effect, innovation agglomeration has a more obvious energy conservation effect. Based on the testing steps and judgment criteria of mediating effect described above, it can be seen from model 4, 15, and 16 that the coefficient of innovation agglomeration is significant in model 15, while its coefficient decreases in the comparison between model 16 and 4. It is proved that energy intensity is the mediating variable of innovation agglomeration affecting carbon emission intensity. Thus, Hypothesis 3 is valid. At the same time, the testing process of energy intensity intermediary effect mentioned above verifies that innovation agglomeration will show the dual effect of saving energy and reducing emission when it reaches the threshold.

By comparing the coefficients of the last two columns in Table 5, it can be seen that there is a completely same influence direction of all control variables on energy intensity and carbon emission, in spite of the slight differences in significance level. Among them, the estimated coefficients of labor productivity, urbanization, energy consumption structure, industrial structure, and marketing degree are all significantly positive. This indicates that these variables simultaneously promote the enhancement in carbon emission intensity and energy intensity. In the meantime, per capita GDP is found to have a negative relationship with both variables. In addition, the coefficients of environmental regulation and opening up are both negative, indicating that the improvement of China’s opening up and environmental regulation intensity have a certain effect in restraining the increase of these two variables in recent years.

## 5. Conclusions and Suggestion

(1) The time lag terms of carbon emission intensity and energy intensity are significant at 1%, and the spatial correlation coefficient is also significant, which indicate that energy intensity and carbon emission intensity have strong spatio-temporal dependence effect. To be more specific, these two variables not only show path-dependence in the dimension of time, but also produce significant spatial spillover effect. For instance, if these two variables in the last period are at a high level, then in the next period they may also continue to rise. It indicates that once the industrial path and technological path are formed, great efforts may be required to change the evolutionary path of carbon emission. Therefore, there is still a long and tough way for the implementation of carbon emission reduction. In the spatial dimension, the carbon emission intensity and energy intensity among regions show a significantly positive spatial correlation, suggesting that China’s current work in terms of carbon emission reduction must be implemented through constructing cooperative linkage effect among regions. It is necessary to strengthen regional cooperation by establishing a joint prevention and control mechanism across administrative regions, forming a coordinated pollution control system in various regions, and clarifying environmental pollution control responsibilities.
(2) Innovation agglomeration may have dual effect of saving energy and reducing emission and the critical value of innovation agglomeration’s emission reduction effect (5.0100) is larger than that of energy conservation effect (3.0309). There is a significant inverted U-shaped curve relationship between innovation agglomeration and carbon emission intensity. When the logarithm of innovation agglomeration exceeds 5.0100, it will have a significant effect on carbon emission reduction. Similarly, there is also a significant inverted U-shaped curve relationship between innovation agglomeration and energy intensity. Therefore, when the level of innovation agglomeration is low, agglomeration will promote the increase of energy intensity. When the logarithm of innovation agglomeration exceeds 3.0309, it can show significant inhibition effect on energy intensity, that is, innovation agglomeration has energy conservation effect. At present, the degree of innovation agglomeration in some developed provinces in China has already passed the critical point, above which innovation agglomeration produces the effect of saving energy and reducing emission. Hence, it could be expected that with the continuous improvement of regional innovation agglomeration level in China, the effect of technological innovation in saving energy and reducing emission will be realized in a larger scope. In China’s inshore areas, there is a significant inverted U-shaped curve relationship between innovation agglomeration and energy intensity or carbon emission intensity. However, in inland areas, innovation agglomeration does not appear to have a significant non-linear effect on carbon emissions, limited to the left part of the inverted U-shaped curve. That is, innovation agglomeration acted as a role in promoting the carbon emission intensity of inland areas. In the meantime, energy intensity has a non-linear positive promotion effect on carbon emissions. Accordingly, more attention should be paid to inshore areas, because they may contribute more to reducing the level of carbon emission in China. We need to adjust the regional innovation system, strengthen cooperation between inshore and inland areas, and facilitate the interconnection and sharing of innovative information resources.
(3) There are direct and indirect paths on the mechanism for innovation agglomeration to affect carbon emission. When the logarithm of energy intensity exceeds 0.8536, carbon emission will be inhibited, which will have a significantly negative correlation between them. Therefore, on the one hand, innovation agglomeration can directly reduce carbon emission intensity through its various positive externalities. On the other hand, innovation agglomeration influences carbon emission through energy intensity, that is, energy intensity acts as its intermediary variable. The specific action mechanism is as follows: When the level of innovation agglomeration is low, it will promote the increase of energy intensity and reduce energy intensity, thus contributing to the decline of carbon emission intensity. As for industrial enterprises, they should be guided to improve production technology and promote energy intensive utilization. We should encourage enterprise to develop green manufacturing industries such as energy conservation and environmental protection industries, new energy equipment, and new energy vehicles, to drive the green upgrading and transformation of industry.
(4) Compared with other studies, the conclusion in this paper is consistent with the researches of Lin et al. (2013) [82] and Zhang et al. (2017) [83], which show that China’s carbon emission intensity has a strong spatio-temporal dependence effect. However, differently from the studies of Li et al. (2020) [28] and Xue et al. (2020) [29], our empirical results show that in China’s inland areas, innovation increases the carbon emissions, while for the whole of China, the relationship between innovation and carbon emissions is nonlinear. Meanwhile, similar to Feng et al. (2019) [84], there is a threshold effect between technological innovation and carbon emissions. The difference is that our paper not only tests the threshold effect between innovation agglomeration and carbon emission intensity, but also empirically studies the nonlinear relationship with energy intensity, showing an energy saving effect of innovation agglomeration. Although this research has made some valuable achievements, it also has some limitations. Firstly, carbon emission intensity is calculated following the provincial data of China. It could be more accurate to use urban data to describe carbon emission intensity in China and the corresponding spatial correlation. Secondly, this paper uses regional patent data to calculate innovation agglomeration by output density model. However, innovation agglomeration includes not only innovation output, but also the aggregation of talents and R&D funds in specific regions, which is the direction that can be expanded in future research.

## Figures and Tables

**Table 1:** Influences of innovation agglomeration and energy intensity on carbon emission intensity under different external conditions.

Variable Name	External	Stage of Development	Environmental Effect	Main Mechanisms
Innovation agglomeration	1 < *λ* < 1/*αl*	Initial stage; Acceleration stage	Promotion	Pursuing economic interests; Lax environmental regulation
	1 < *α* < 1/*αl*	Mature stage	Inhibition	Green technology spillover; Economies of scale; Resetting technological trajectory; Greening industrial structure
Energy intensity	1 < *λ* < 1/*αl*	Initial stage; Acceleration stage	Promotion	Increasing demand for energy; Outdated energy conservation Technologies; Lax environmental regulation
	1 < *α* < 1/*αl*	Mature stage	Inhibition	Reasonable energy allocation structure; Clean energy structure;

**Table 2:** Data Selection & Description.

Variable Types	Index Selection	Symbol	Indicator Description	Variable Source
Explained variable	Carbon emission	*CO_2_*	Carbon emission from non-farm	China Energy Statistics Yearbook (2005–2018)
Core explanatory variable	Innovation agglomeration	*Agin*	Number of patents granted /administrative area	China Science and Technology Statistical Yearbook (2005–2018)
	Energy intensity	*Sen*	Energy consumption/industrial value-added	China Energy Statistics Yearbook (2005–2018)
Control variables	Labor productivity	*Ip*	Industrial output/employment	China Statistical Yearbook (2005–2018) China Science and Technology Statistical Yearbook (2005–2018) China Environmental Yearbook (2005–2018)
	Per capita income level	*Pcin*	GDP/population	
	urbanization	*Ur*	Urban population/total population	
	Energy consumption structure	*Es*	Coal consumption/energy consumption	
	Industrial structure	*Ind*	Industrial value-added/GDP	
	Opening up	*Open*	Total imports and exports/GDP	
	Environmental regulation	*Er*	Environmental expenditure/GDP	
	Marketing	*Mar*	Marketization index	Marketization Index Report

**Table 3:** Spatial Correlation Test.

Model Type	Static Durbin			Dynamic Durbin		
Model	3	13	14	4	15	16
Global Moran’s I [P]	0.2308 *** (0.000)	0.7664 *** (0.000)	0.2480 *** (0.000)	0.6665 *** (0.000)	0.7843 *** (0.000)	0.7412 *** (0.000)
LM-lag [P]	29.8825 *** (0.000)	4.1013 ** (0.042)	35.0508 *** (0.000)	19.6130 *** (0.000)	21.5171 *** (0.000)	36.154 *** (0.000)
LM-error [P]	29.8802 *** (0.000)	5.1024 ** (0.023)	35.0545 *** (0.000)	244.2844 *** (0.000)	349.7951 *** (0.000)	318.7048 *** (0.000)

Note: *** and ** representing the significance level of 1% and 5%, respectively.

**Table 4:** Impact of Agin and Sen on carbon emission intensity.

Variable	OLS-FE	SYS-GMM	Static Durbin	Dynamic Durbin
Model	1	2	3	4
*L.CO_2_*		0.4630 *** (5.08)		0.3618 *** (2.90)
*Agin*	0.2648 *** (4.80)	0.0835 (0.27)	0.2119 *** (4.11)	0.3498 *** (4.01)
*sAgin*	−0.0388 *** (−4.32)	−0.0135 (−0.27)	−0.0169 * (−1.96)	−0.0462 *** (−3.03)
*Sen*	0.1332 ** (2.43)	0.1405 *** (2.92)	0.1837 *** (3.59)	0.1549 ** (1.99)
*sSen*	−0.0113 (−0.22)	−0.0117 (−0.23)	−0.0111 ** (−2.53)	−0.0126 ** (−2.17)
*Lp*	0.2170 * (1.69)	0.4042 (1.36)	0.3286 *** (2.66)	0.4020 ** (2.43)
*Eco*	0.8076 (1.24)	0.6237 (0.98)	0.6714 (1.06)	0.8919 ** (2.53)
*sEco*	−0.0033 (−0.11)	−0.0292 −(0.44)	−0.0606 ** (−2.04)	−0.1013 * (−1.88)
*Ur*	0.5703 (1.07)	0.5314 (0.96)	0.1632 * (1.77)	0.4041 * (1.76)
*sUr*	0.3674 (1.34)	0.5088 (0.54)	0.2009 (0.08)	0.2016 ** (2.00)
*Es*	0.2102 *** (4.44)	0.0971 ** (2.22)	0.2401 *** (5.13)	0.2191 *** (5.33)
*Ind*	0.0984 (1.56)	0.2384 ** (2.07)	0.2276 *** (3.78)	0.0802 * (1.88)
*Open*	−0.0319 *** (−2.21)	−0.0637 *** (−2.19)	−0.0349 (−1.41)	−0.0786 ** (−2.37)
*Er*	−0.0429 ** (−1.97)	−0.0603 ** (−2.58)	−0.0408 ** (−2.31)	−0.0577 ** (−2.21)
*Mar*	0.4414 *** (5.29)	0.7056 *** (7.14)	0.0813 *** (4.37)	0.4139 *** (3.65)
*Cons*	2.4114 (0.72)	−7.2394 (−0.77)	5.9700 * (1.86)	3.1693 (0.44)
*F(Wald)* *[P]*	164.52 (0.00)	174.64 (0.00)	3065.36 (0.00)	933.27 (0.00)
*AR(2)*		1.1386		0.6563
*Sargan[P]*		27.2539 (0.9997)		26.8437 (0.9998)
*Log*			362.0113	218.7573
*Rho*			0.3184 *** (4.14)	2.5591 *** (5.75)
*Obs*	420	390	420	390

Note: The values in brackets are T-value or Z-value, with ***, **, and * representing the significance level of 1%, 5%, and 10%, respectively. *sAgin* is the square term of innovation agglomeration; *sSen* is the square term of energy intensity; *L.CO_2_* is the time lag term of the logarithm of carbon emission intensity; Spatial dynamic panel model and non-spatial dynamic panel model (SYS-GMM) reported *Wald test* results, other models reported *F-test* results; *AR(2)* and *sargan texts* show the GMM estimation method is reasonable; the following tables are the same.

**Table 5:** Regional test based on the spatial Durbin model.

Variable	Static Durbin		Dynamic Durbin	
	Inshore	Inland	Inshore	Inland
Model	5	6	7	8
*L.CO_2_*			0.9904 *** (7.81)	0.5082 *** (5.64)
*Agin*	3.36388 *** (2.66)	0.2914 *** (4.09)	2.4505 *** (3.11)	0.2231 *** (3.50)
*sAgin*	−0.4753 *** (−2.92)	−0.0096 (−0.64)	−0.2696 *** (−2.73)	−0.0076 (−0.59)
*Sen*	0.1703 (0.50)	0.1440 ** (2.13)	0.9114 *** (5.20)	0.2248 *** (3.66)
*sSen*	−1.1120 *** (−3.16)	0.0748 (1.53)	−0.2696 *** (−2.73)	0.2200 *** (3.98)
*Lp*	1.4830 ** (2.57)	0.4170 *** (2.85)	1.1699 *** (3.60)	0.4493 *** (2.96)
*Eco*	0.9276 *** (4.34)	0.0103 (0.01)	0.9447 *** (3.90)	0.2053 (1.48)
*sEco*	−0.0595 *** (−4.63)	−0.0464 (−0.99)	−0.4814 *** (−4.62)	−0.2952 (−0.81)
*Ur*	0.3033 *** (4.34)	0.3959 (0.53)	1.2274 (1.32)	0.4504 (0.69)
*sUr*	0.0595 (4.63)	0.2262 (0.60)	0.7340 * (1.88)	0.0324 (0.31)
*Es*	0.5864 *** (3.20)	0.0742 (0.92)	0.2773 ** (2.09)	0.0192 (0.15)
*Ind*	0.0550 *** (3.61)	0.0704 ** (0.68)	1.1550 *** (4.97)	0.0802 * (1.88)
*Open*	−0.4801 ** (−2.01)	−0.0683 ** (−2.15)	−0.7651 *** (−6.72)	−0.0123 (−0.28)
*Er*	−0.0095 (−0.15)	−0.0410 (−1.40)	−0.0257 ** (−2.31)	−0.0509 (−1.42)
*Mar*	0.4753 *** (4.08)	0.5238 *** (4.44)	1.6531 *** (4.36)	0.5308 *** (3.36)
*Cons*	2.4114 (0.72)	3.1624 (0.33)	−37.0666 *** (−3.28)	−10.1274 (−0.94)
*F(Wald)* *[P]*	164.52 (0.00)	174.64 (0.00)	865.36 (0.00)	706.66 (0.00)
*AR(2)*			0.7115	0.7654
*Sargan* *[P]*			28.4182 (0.9995)	27.2240 (0.9876)
*Rho*	0.3225 *** (4.58)	0.1028 ** (2.36)	2.8445 *** (6.78)	2.0112 *** (3.38)
*Log*	124.4714	169.7098	132.5438	159.5702
*Obs*	154	266	143	247

Note: The values in brackets are T-value or Z-value, with ***, **, and * representing the significance level of 1%, 5%, and 10%, respectively.

**Table 6:** Energy intermediation effect test based on carbon emission intensity.

Variable	OLS-FE		SYS-GMM		Static Durbin		Dynamic Durbin	
	L.Sen	L.CO_2_	L.Sen	L.CO_2_	L.Sen	L.CO_2_	L.Sen	L.CO_2_
Model	9	10	11	12	13	14	15	16
*L.CO_2_*				0.3728 *** (5.13)				0.4857 *** (3.47)
*L.Sen*			0.9253 *** (29.78)				0.7690 *** (3.88)	
*Agin*	0.1001 * (1.74)	0.2743 *** (4.57)	0.1292 ** (2.25)	0.1997 ** (2.53)	0.0440 * (1.87)	0.2287 *** (3.81)	0.1334 * (1.92)	0.4609 *** (4.77)
*sAgin*	−0.0334 *** (−3.68)	−0.0362 *** (−4.45)	−0.0259 *** (−2.93)	−0.0315 ** (−2.29)	−0.0233 ** (−2.47)	−0.0151 *** (−2.41)	−0.0357 *** (−4.08)	−0.0228 *** (−2.63)
*Lp*	0.3005 ** (2.21)	0.2817 ** (2.13)	0.1603 (1.55)	0.4919 (1.16)	0.1737 (1.25)	0.3941 *** (3.08)	0.1176 * (1.95)	0.2821 ** (2.31)
*Eco*	2.7502 *** (4.07)	1.2542 * (1.91)	0.8761 (1.20)	0.1129 (0.712)	2.2406 (3.32)	0.0264 (0.04)	1.6020 ** (2.26)	0.347 (0.62)
*sEco*	−0.1359 *** (−4.24)	−0.0260 (−0.84)	0.1802 (0.46)	0.0306 (0.79)	−0.1088 ** (−2.57)	−0.0265 (0.87)	−0.0756 ** (−2.25)	−0.0266 (−1.40)
*Ur*	1.1311 *** (2.00)	0.8794 (1.61)	0.1300 (0.20)	2.240* (1.91)	1.2511 ** (2.57)	0.5657 (1.14)	1.0111 ** (2.01)	0.5967* (1.89)
*sUr*	0.7325 ** (2.54)	0.5826 ** (2.09)	0.1802 (0.46)	0.9450 (1.16)	0.6778 ** (2.57)	0.2419 (0.94)	0.6920 ** (2.58)	0.2529 (0.54)
*Es*	0.0286 (0.43)	0.2458 *** (5.30)	0.0575 (0.23)	0.1425 *** (3.14)	0.2024 *** (4.19)	0.2764 *** (6.21)	0.9584 ** (2.20)	0.2184 *** (5.08)
*Ind*	−0.0200 (−0.44)	0.1327 ** (2.06)	0.0703 (1.50)	0.4197 *** (3.69)	0.0516 (0.74)	0.2392 *** (3.85)	0.2148 *** (3.04)	0.0663 ** (2.53)
*Open*	−0.0232 (−0.85)	0.2182 *** (4.90)	−0.0349 ** (−2.50)	−0.071 6*** (−6.69)	−0.0631 ** (−2.32)	−0.0334 (−1.33)	−0.0376 * (−1.71)	−0.0337 *** (−2.37)
*Er*	−0.0003 (−0.01)	−0.0424 * (−1.89)	−0.0181 ** (−2.56)	−0.5331 ** (−2.46)	−0.0423 * (−1.75)	−0.0302 (−1.40)	−0.0051 (−0.24)	−0.0386 * (−1.89)
*Mar*	0.1993 ** (2.27)	0.5090 *** (5.98)	0.1050 *** (4.09)	0.7751 *** (8.82)	0.0247 (0.28)	0.4119 (4.92)	0.2739 *** (2.92)	0.2829 *** (4.59)
*Cons*	−13.032 *** (−3.72)	0.4936 (0.15)	4.8002 (1.27)	1.6620 (0.30)	−10.3551 ** (−2.29)	2.9768 (0.91)	−9.1185 (−1.21)	2.2134 (0.29)
*F(Wald)* *[P]*	69.78 (0.00)	185.58 (0.00)	218.76 (0.00)	282.88 (0.000)	907.63 (0.00)	923.12 (0.00)	882.12 (0.00)	858.53 (0.00)
*AR(2)*			0.9698	1.0845			0.8760	0.7665
*Sargon* *[P]*			15.6689 (0.8690)	25.3948 (0.9864)			27.2341 (0.9997)	26.3129 (0.9998)
*Rho*					0.1640 ** (2.03)	0.3763 *** (4.93)	2.5743 * (1.85)	2.1680 *** (5.38)
*Log*					299.3801	312.3365	256.8333	253.4485
*Obs*	420	420	390	390	420	420	390	390

Note: The values in brackets are T-value or Z-value, with ***, **, and * representing the significance level of 1%, 5%, and 10%, respectively.

## Data Availability

No new data were created or analyzed in this study. Data sharing is not applicable to this article.
